# Elevated Urine Heparanase Levels Are Associated with Proteinuria and Decreased Renal Allograft Function

**DOI:** 10.1371/journal.pone.0044076

**Published:** 2012-09-13

**Authors:** Itay Shafat, Amir Agbaria, Mona Boaz, Doron Schwartz, Ronny Baruch, Richard Nakash, Neta Ilan, Israel Vlodavsky, Talia Weinstein

**Affiliations:** 1 Cancer and Vascular Biology Research Center, the Bruce Rappaport Faculty of Medicine, Technion, Haifa, Israel; 2 Department of Nephrology, Tel Aviv Medical Center, Tel Aviv, Israel; 3 Epidemiology Unit, E. Wolfson Medical Center, Holon, Israel; 4 Transplant Clinic, Tel Aviv Medical Center, Sackler School of Medicine, Tel Aviv University, Tel Aviv, Israel; Universidade de Sao Paulo, Brazil

## Abstract

Heparanase is an endo-β-glucuronidase that cleaves heparan sulfate side chains, leading to structural modifications that loosen the extracellular matrix barrier and associated with tumor metastasis, inflammation and angiogenesis. In addition, the highly sulfated heparan sulfate proteoglycans are important constituents of the glomerular basement membrane and its permselective properties. Recent studies suggest a role for heparanase in several experimental and human glomerular diseases associated with proteinuria such as diabetes, minimal change disease, and membranous nephropathy. Here, we quantified blood and urine heparanase levels in renal transplant recipients and patients with chronic kidney disease (CKD), and assessed whether alterations in heparanase levels correlate with proteinuria and renal function. We report that in transplanted patients, urinary heparanase was markedly elevated, inversely associated with estimated glomerular filtration rate (eGFR), suggesting a relationship between heparanase and graft function. In CKD patients, urinary heparanase was markedly elevated and associated with proteinuria, but not with eGFR. In addition, urinary heparanase correlated significantly with plasma heparanase in transplanted patients. Such a systemic spread of heparanase may lead to damage of cells and tissues alongside the kidney.The newly described association between heparanase, proteinuria and decreased renal function is expected to pave the way for new therapeutic options aimed at attenuating chronic renal allograft nephropathy, leading to improved graft survival and patient outcome.

## Introduction

The glomerular capillary barrier exerts both size- and charge-selective properties [1]. The charge-selective feature is attributed primarily to highly sulfated proteoglycans which reside in the glomerular basement membrane (GBM), podocytes, and the glomerular endothelium [2], [3]. Of particular significance are heparan sulfate (HS) proteoglycans (HSPGs), consisting of a core protein to which glycosaminoglycan HS chains are attached and held, among other factors, responsible for the permselective properties of the glomerular capillary wall. Loss of HSPGs was observed in several experimental and human glomerulopathies, including diabetic nephropathy, minimal change disease, and membranous glomerulopathy [4], [5], where a decrease in HS inversely correlates with proteinuria [6]–[9]. Accordingly, administration of monoclonal anti-HS antibody resulted in massive proteinuria in a rat model [10], and removal of HS by enzymatic cleavage resulted in increased GBM permeability [6], supporting a contribution of HS to glomerular permselectivity. Decreased content of HS has been noted in the glomerular barrier in a variety of human and experimental proteinuric diseases, attributed, in part, to over-expression of heparanase [5], [11]–[16].

Heparanase is an endo-β-glucuronidase that cleaves HS side chains of HSPGs presumably at sites of low sulfation, leading to disassembly of the ECM and BM, thereby affecting fundamental biological phenomena associated with cell motility and tissue remodeling [17]–[20]. Expression of heparanase, the only mammalian endoglycosidase that degrades HS side chains, was up-regulated in animal models of proteinuric renal disease including passive Heymann nephritis [21], puromycinnephrosis [14], anti-GBM nephritis [22], and adriamycin nephropathy [23], likely damaging the permselective properties of HS. Indeed, over-expression of heparanase in transgenic mice leads to proteinuria [24], while treatment with a polyclonal anti-heparanase antibody resulted in a 3-fold reduction of proteinuria in a model of anti-GBM disease [22]. Similarly, PI-88, a sulfated oligosaccharide inhibitor of heparanase, significantly reduced the loss of glomerular HSPGs and the associated proteinuria [13], further emphasizing the involvement of heparanase in the development of proteinuria.

Since heparanase activity is associated with a loss of glomerular HS and consequent proteinuria, the present study was undertaken to determine plasma and urine heparanase levels in renal transplant recipients and chronic kidney disease (CKD) patients and to assess whether alterations in heparanase levels correlate with proteinuria and kidney function.We report that urinary heparanase is markedly elevated in patients with CKD and following kidney transplantation. Notably, urine heparanase was significantly associated with proteinuria and inversely associated with estimated glomerular filtration rate (eGFR) in transplanted patients. A highly significant association was found between urine and plasma heparanase levels in transplanted patients, suggesting that heparanase is present systemically and can affect cells and tissues other than the kidney. Heparanase inhibitors may thus protect the kidney and improve its function in transplanted patients.

## Subjects and Methods

### Patients

Eligible renal transplant recipients followed at the Tel Aviv Medical Center Transplant Clinic were recruited by their nephrologists. Transplant patients were recruited from attendees at the post-transplant clinic. This convenience sample was developed by recruiting the first 100 patients attending clinic on consecutive days using the following criteria: adult patients aged 18 years and older with stable renal function for at least three months prior to study commencement; no evidence of urinary tract infection or other systemic disease; and no signs of acute rejection or glomerulonephritis. Recruitment was completed within three weeks. All patients approached agreed to participate and signed a formed consent. CKD patients are a convenience sample of attendees at the Nephrology Clinic. Patients attending clinic during a two week period were approached and asked for an additional test. All 41 patients approached agreed and signed informed consent. Healthy controls were recruited from the clinic staff.

The transplanted patients enrolled were diagnosed as type 1 (n = 21) and type 2 (n = 13) diabetes, adult polycystic kidney disease (n = 17), chronic glomerulonephritis (n = 6), nephrosclerosis (n = 6), focal segmental glomerulosclerosis (n = 5), IgA nephropathy (n = 2), systemic lupus (n = 2), nephrolithiasis (n = 2), reflux nephropathy (n = 2), membranoproliferative glomerulonephritis (n = 1), membranous nephropathy (n = 1), fibrillary glomerulonephritis (n = 1), and patients with end stage kidney disease with unknown etiology (n = 21).Ninety-one patients were treated for hypertension. The patients had undergone renal transplantation one to ten years earlier and were treated with a conventional immunosuppressive protocol including corticosteroids, calcineurin inhibitors, and azathioprine or mycofenolatemofetil. Seven patients were treated with cyclosporine, maintaining a trough level between 100–120 ng/ml, one patient was treated with sirolimus, and the other 92 patients were treated with tacrolimus, maintaining trough levels between 4–8 ng/ml.

CKD patients enrolled included type 1 and type 2 diabetes (1 and 20 patients, respectively), atherosclerotic vascular disease (n = 6), hypertension (n = 10), nephrolithiasis (n = 2), focal segmental glomerulosclerosis (n = 1), and systemic lupus erythematosus (n = 1).

Morning blood was drawn for biochemistry, complete blood count and heparanase content. Fresh morning urine samples were obtained for measurements of protein, albumin, creatinine and heparanase. All blood and urine samples for heparanase assay were placed immediately on ice; plasma was separated by centrifugation (5 min, 1200 g, 4°C), and samples were kept at −70°C until analyzed. All patients signed informed consent to participate in the study, which was approved by the Tel Aviv Medical Center Committee for Studies in Human Beings.

### Laboratory measurements

Blood and urine chemistry, including creatinine, protein, and albumin levels were determined using Advia 1650 equipment (Siemens). Blood count was performed by LH Beckman Coulter. Estimated glomerular filtration rate (eGFR) was determined by the abbreviated four-variable Modification of Diet in Renal Disease (MDRD) equation [26]. Urinary protein and urinary albumin levels are presented as milligram protein or albumin per gram creatinine.

### Heparanase determination

Heparanase levels were determined according to a previously described ELISA method [25], [27], [28]. Briefly, wells of microtiter plates were coated (18 h, 4°C) with 2 µg/ml 1E1 anti-heparanase monoclonal antibody in 50 µl of coating buffer (0.05 M Na_2_CO_3_, 0.05 M NaHCO_3_, pH 9.6) and were then blocked with 1% BSA in PBS for 1 h at 37°C. Samples (200 µl) were loaded in duplicates and incubated for 2 h at room temperature, followed by the addition of 100 µl anti-heparanase polyclonal antibody 1453 (1 µg/ml) for additional 2 h at room temperature. HRP-conjugated goat anti-rabbit IgG (Jackson ImmunoResearch, West Grove, PA; 1∶20,000) in blocking buffer was then added (1 h, room temperature) and the reaction was visualized by the addition of 100 µl chromogenic substrate (TMB) for 30 min. The reaction was stopped with 100 µl H_2_SO_4_ and absorbance at 450 nm was measured with reduction at 630 nm using ELISA plate reader. Plates were washed (×5) with washing buffer (PBS, pH 7.4, containing 0.1% (v/v) Tween 20) after each step. As a reference for quantification, a standard curve was established by a serial dilution of recombinant active heparanase enzyme (25 ng/ml–390 pg/ml), as described [25], [27], [28]. Urine heparanase levels are expressed as nano gram heparanase per gram of urinary creatinine.

## Statistical Methods

### Data analysis

Data analysis was carried out using SPSS version 11.0 statistical analysis software (SPSS Inc., Chicago, IL, USA). Continuous variables are reported as mean ± standard deviation. Variables with distributions significantly deviating from normal are described in addition as median (min-max). Distributions of continuous variables were assessed for normality using the Kolmogorov-Smirnov test (cut off at p = 0.01). One-way analysis of variance (ANOVA) or the Kruskal-Wallis test were used to compare continuous variables across patient category (transplanted, CKD or control), followed post hoc by Bonferroni's pair wise analysis or the Mann-Whitney U-test, as appropriate. Associations between continuous variables were determined by calculating the Pearson's or Spearman's rho correlation coefficients. In transplant patients, eGFR was modeled using multiple linear regression analysis. The most parsimonious model was achieved using a backward approach, with a probability of F at 0.05 for entry and 0.1 for removal. In transplant patients, eGFR was modeled using multiple linear regression analysis. The most parsimonious model was achieved using a backwards, stepwise approach. Categorical variables such as sex were described using frequency distributions (n %) and compared across patient group using the chi square test. All tests are two-sided and considered significant at p<0.05.

## Results

### Clinical parameters

We applied an ELISA method to determine heparanase levels in plasma and urine of patients with CKD and patients who underwent kidney transplantation compared to control healthy volunteers.Clinical and demographic description of recruited patients is shown in Table 1. Notably, the demographic characteristics of the patients recruited for the study mirrored the transplant population (n = 237) in our clinic in terms of gender (38% females), age (52.9±12.9 years), and serum creatinine (1.62±0.6 mg/dl). The study group therefore highly represents kidney transplant patients at the Tel Aviv Medical Center.

**Table 1:** Characteristics of study population by diagnostic group.

Parameter	Controls (n = 18)	CKD (n = 41)	Transplant (n = 100)	p-value^a^
**Age**	46.1±10.6	73.7±13.2	51.2±12	
**Sex (% female)**	44	32	35	
**eGFR (ml/min)** b	75.7±2.7	30±2.2	53.7±1.7	<0.0001
**Serum creatinine (mg/dL)**	1.0±0.1	2.6±0.1	1.4±0.1	<0.0001
**Urinary albumin/creatinine (mg/gr; median)**	13.1 (7–24)	281.1 (11–4674)	23.3 (2–13978)	0.001
**Urinary protein/creatinine (mg/gr; median)**	69.7 (40–140)	585.1 (110–7460)	195.1 (50–5820)	0.001
**Serum albumin (g/L)**	44.7±1	40.2±0.6	43.4±3.4	0.001
**Serum protein (g/L)**	72.4±2	70.4±0.8	71.1±0.5	0.2
**Hemoglobin (g/dL)**	14.3±0.6	11.9±0.2	13.6±0.2	<0.0001
**Plasma heparanase (pg/ml)**	71.7±22	136.9±24	193.9±73	<0.0001
**Urinary heparanase/creatinine (ng/gr; average, median)**	307.2±95.1 130.3(16–1520)	950.3±155.9 631.1(148–4718)	1008±134.0 625(0–9775)	<0.0001

Data is presented as mean±S.D and/or as median values (min-max).

a One-way analysis of variance (ANOVA) or the Kruskal-Wallis test were used to compare continuous variables across patient category (control, CKD and transplanted), followed post hoc by Bonferroni's pair wise analysis or the Mann-Whitney U-test, as appropriate.

b Estimated glomerular filtration rate (eGFR) was determined by the abbreviated Modification of Diet in Renal Disease (MDRD) equation [26].

Urinary albumin/creatinine levels were strikingly increased in CKD, and to a lesser extent in transplanted patients (median values of 13.1, 281.1, and 23.3 mg/gr for control, CKD and transplanted patients, respectively; Table 1). A similar trend was noted for urinary protein/creatinine levels (69.7, 585.1, 195.1 mg/gr in average for control, CKD and transplanted patients, respectively; Table 1). The levels of serum albumin, hemoglobin and eGFR were decreased in CKD patients but were higher in patients following kidney transplantation (Table 1). An inverse association between eGFR and proteinuria (p = 0.005), and between eGFR and albuminuria (p = 0.027) was noted in CKD patients, as expected. Hemoglobin was significantly associated with eGFR (r = 0.293, p = 0.004, Table 1) likely since a healthier allograft produces more erythropoietin, altogether indicating that the transplanted kidney is functioning.

### Heparanase elevationin CKD and transplanted patients

**Figure 1:**
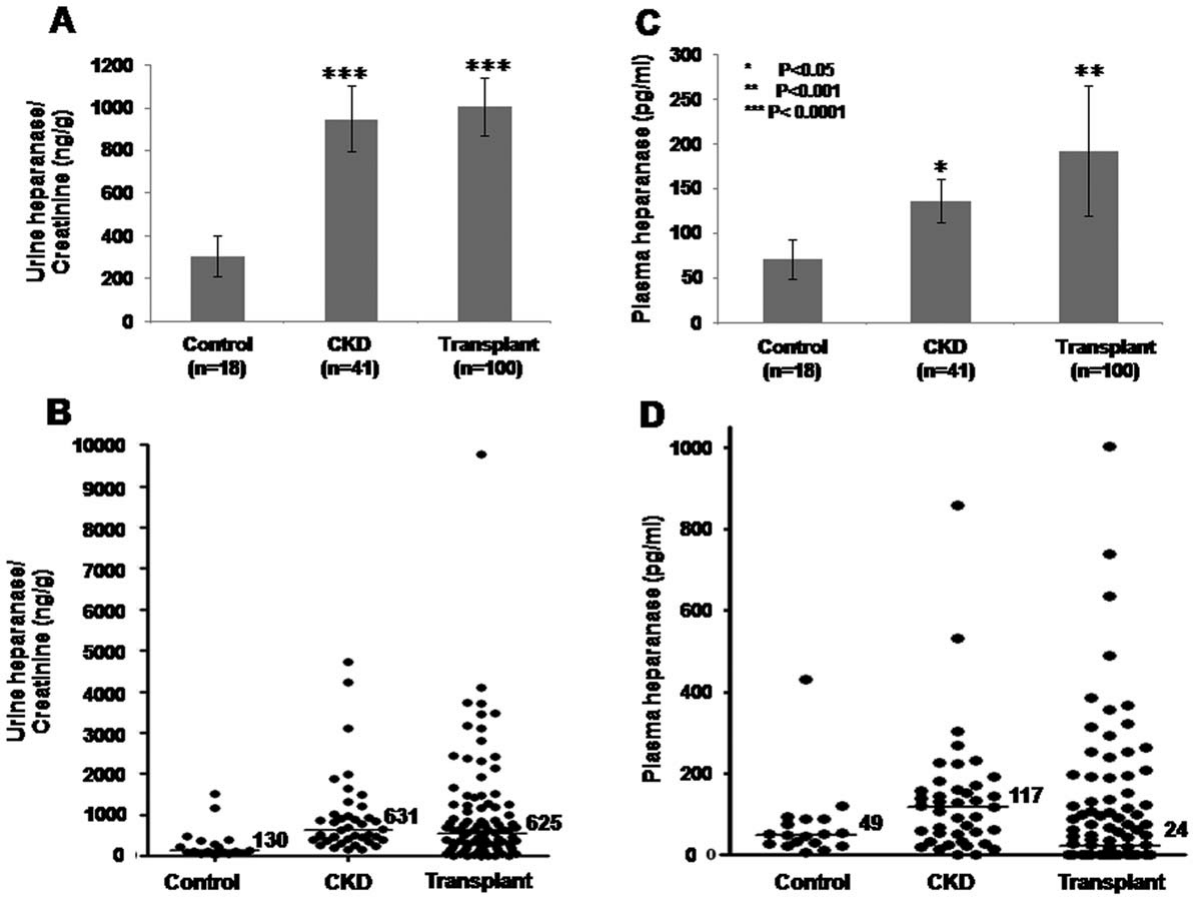
Heparanase levels in the urine and blood of study subjects. Determination of heparanase levels in urine (A, B) and plasma (C, D) of individuals from the study groups. Shown are average (±SE; A, C) and median (B, D) values quantified by an ELISA method, as described under ‘Materials and Methods’.

Heparanase/creatinine levels were elevated three- and four-fold in the urine of CKD and transplanted patients (307.2±95, 950±155, and 1008±134 ng heparanase/gr creatinine in average for control, CKD, and transplanted patients, respectively; Fig. 1A, Table 1), an elevation that is highly significant (p<0.0001 for control vs. CKD and control vs. transplanted patients; Fig. 1A, Table 1). Similar elevation of urine heparanase in CKD and transplanted patients is found once median values are plotted (Fig. 1B). Elevation of heparanase, albeit lower in magnitude, was quantified also in the plasma of CKD and transplanted patients (71.7±22, 136.9±24, and 193.9±73 pg/ml in average for control, CKD and transplanted patients, respectively; Fig. 1C, Table 1) (p<0.05 for control vs. CKD and p<0.01 for control vs. transplanted patients; Fig. 1C). Once median values are calculated, however, plasma heparanase is increased in CKD patients but decreased to control levels following kidney transplantation (49, 117, and 24 pg heparanase/gr creatinine for control, CKD, and transplanted patients, respectively, Fig. 1D). A highly significant association (p<0.01) was found between urine and plasma heparanase levels in transplanted patients (Table 2), suggesting that heparanase is present systemically and can affect cells and tissues other than the kidney. No such association was found in CKD patients (Table 2).

**Table 2:** Association between log transformed urine heparanase/creatinine levels and studied parameters in transplanted (n = 100) and CKD (n = 41) patients^a^.

	Log transformed urine heparanase/creatinine			
	CKD		Transplant	
Parameter	correlation coefficient	p value	correlation coefficient	p value
**Age**	0.118	0.463	−0.016	0.883
**Years since transplantation**			0.313	0.002
**Plasma heparanase**	0.1	0.54	0.261	0.01
**eGFR** b	−0.01	0.936	−0.223	0.03
**Urinary protein/creatinine**	0.459	0.001	0.284	0.006
**Urinary albumin/creatinine**	0.513	0.003	0.147	0.3
**Serum albumin**	−0.263	0.097	−0.243	0.02

a Pearson's or Spearman's correlation coefficients are presented within CKD and transplant groups, respectively.

b Estimated glomerular filtration rate (eGFR) was determined by the abbreviated Modification of Diet in Renal Disease (MDRD) equation [26].

**Figure 2:**
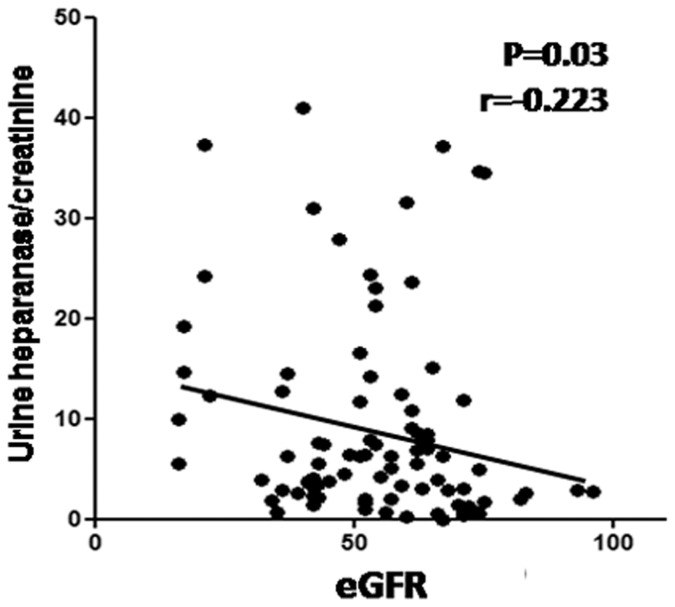
Urine heparanase levels inversely associate with eGFR in kidney transplanted patients. Heparanase/creatinine levels are plotted in relation to eGFR values. Note significant inverse association of urine heparanase with eGFR (p = 0.035, r = −0.2224) (Spearman nonparametric correlation test).

We next examined association of plasma and urine heparanase levels with clinical parameters. In transplanted patients, urine heparanase/creatinine was significantly associated with urine protein/creatinine (p<0.006; Table 2), and significantly inversely associated with serum albumin (p<0.02; Table 2), suggesting a causal effect of heparanase in the development of proteinuria and hypoalbuminemia. Notably, an inverse association was found between urine heparanase/creatinine and eGFR (p = 0.03; Table 2; Fig. 2). Moreover, urine heparanase/creatinine positively associated with the time (years) since transplantation (r = 0.313, p = 0.002; Table2), suggesting that elevated heparanase levels may lead to dysfunction of the grafted kidney. Notably, both urine albumin/creatinine and urine protein/creatinine ratios were associated with urinary heparanase/creatinine in CKD patients (p = 0.003, p = 0.001, respectively; Table 2).

**Figure 3:**
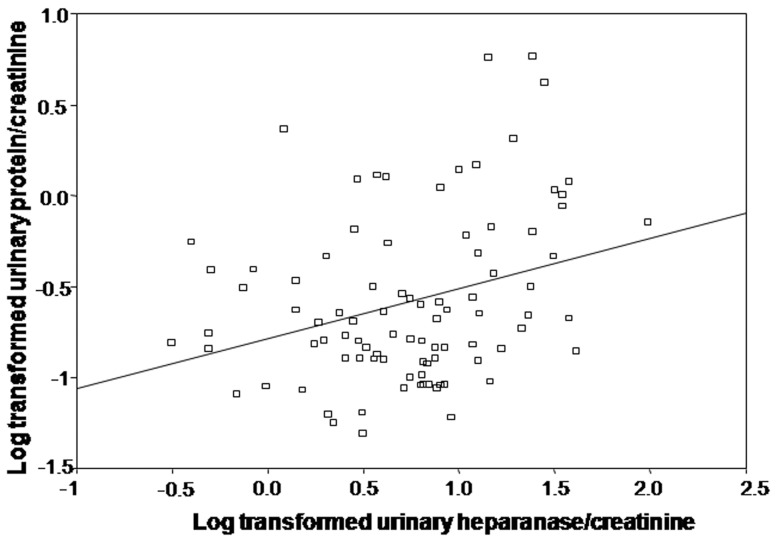
Log transformed urinary heparanase/creatinine and log transformed urinary protein/creatinine.

The association between log transformed urinary heparanase/creatinine and eGFR was modeled in a linear regression analysis. In addition to log transformed urinary heparanase/creatinine ratio, the variables age, years since transplantation, the urinary protein/creatinine ratio as well as interaction terms were included. The final model, which included only log transformed urinary heparanase/creatinine ratio, was significant (beta = −7.9, 95% CI −14.6- −1.3, p = 0.02), but explained only 6% of the variability in eGFR. Similarly, log transformed urinary protein/creatinine was modeled using log transformed urinary heparanase/creatinine ratio as well as age, years since transplantion, eGFR and interaction terms. In the final model, also arrived at using a stepwise, backward approach, only log transformed urinary heparanase/creatinine ratio remained (Fig. 3). Again, while the model was significant (beta = 0.27, 95% CI 0.1–0.5, p = 0.003), it explained only 9% of the variability in log transformed urinary protein/creatinine. Convergence was not reached for a model of urinary albumin/creatinine. In contrast, no association was found between urine heparanase/creatinine ratio and eGFR in CKD patients, suggesting that such a correlation observed in transplanted patients is not solely due to kidney dysfunction or proteinuria. Also, there was no association between urine heparanase/creatinine and plasma heparanase in CKD patients.

## Discussion

Kidney transplantation is the treatment of choice for end-stage renal disease, conferring the highest survival benefit among the different renal replacement therapies. However, many transplanted patients develop proteinuria and chronic allograft dysfunction. The exact mechanism(s) leading to long-term graft loss are unclear. Both immune-dependent and -independent factors are thought to play a role [29]. Despite improved immunosuppressive regimens, chronic allograft nephropathy (CAN) remains the main cause of graft loss. CAN is associated with histologic evidence of fibrosis, proteinuria, and a progressive decrease in eGFR [30]. Proteinuria post transplant can be either secondary to CAN or to recurrent glomerulopathy, or diabetic nephropathy, and we cannot rule out the possibility that some patients in this study may exhibit an additional glomerulopathy.

The present study evaluated heparanase as a potential novel player associated with renal dysfunction in renal transplant recipients. We examined plasma and urine heparanase levels in renal transplant recipients and CKD patients and addressed relevant clinical correlations. Notably, in transplanted patients urinary heparanase/creatinine ratio was significantly, inversely associated with eGFR (Table 2, Fig. 2). Moreover, urine heparanase/creatinine ratio positively associated with the years post transplantation. In contrast, no association was found between urine heparanase/creatinine ratio and eGFR in CKD patients, suggesting that this correlation observed in transplanted patients is not solely due to kidney dysfunction or proteinuria. These findings thus imply that increased urinary heparanase may exert a deleterious effect on renal graft function.

Importantly, we found that urinary heparanase levels correlate significantly with proteinuria in CKD and transplanted patients (Table 2), suggesting a role for heparanase in kidney dysfunction, in agreement with previous pre-clinical and clinical studies [4], [5]. Increased urinary heparanase has also been implicated in several human proteinuric diseases such as diabetes, minimal change disease, membranous nephropathy and IgA nephropathy [5], [31]. A loss of GBM and tubular HS was noted in kidney biopsies from patients with overt diabetes, accompanied by increased expression of heparanase [15], [16]. Development of proteinuria and CKD may be associated with heparanase-mediated damage to the glomerular filtration barrier. It has been well established in CKD that the degree of proteinuria strongly relates to kidney survival. This observation has also been demonstrated in renal transplant recipients where there is a strong association between proteinuria and reduced kidney allograft survival [32]. This relationship extends also to lower levels of proteinuria that are not associated with glomerular pathology. Seventy-seven percent of allografts lost within the first 5 years post-transplant had proteinuria at 1 year [33]. This study and others demonstrate that proteinuria provides an independent prognostic factor for renal graft survival beyond histologic findings. Collectively, these results suggest that heparanase may play a significant role in the pathogenesis of proteinuria and kidney dysfunction.

In addition, urinary heparanase correlated significantly with plasma heparanase levels in transplanted (p = 0.01), but not in CKD (p = 0.54; Table 2) patients. Such a systemic presence of heparanase may lead to the damage of cells and tissues alongside the kidney. In the transplanted kidney, chronic endothelial injury is characterized by the presence of disruption of lamina elastica interna, inflammatory cells in the fibrotic intima and proliferation of myofibroblasts in the intima, indicative of chronic T-cell-mediated rejection [30]. Continuous exposure of the vascular endothelium to heparanase may lead to a severe outcome. It has been shown, for example, that heparanase over-expression leads to arterial thickening and reduced mechanical strength [34]. Moreover, heparanase over-expression enhances neointimal thickening due to increased proliferation and recruitment of macrophages following stent implementation [34], whereas neutralizing anti-heparanase antibodies reduced neointima formation in a rat carotid balloon injury model [35].

Accumulating evidence suggests that heparanase functions also as a pro-coagulation mediator, enhancing expression of tissue factor and generation of factor Xa, two critical components in blood coagulation [36]–[38] thus providing another mode by which heparanase affects the vasculature. Continuous exposure to high levels of heparanase in the circulation may thus contribute to the development of CKD-, and transplantation-associated vasculopathies.

We have reported recently that heparanase levels are elevated in the urine and plasma of diabetic patients [31]. In diabetic patients, heparanase levels were significantly reduced following kidney transplantation implying that heparanase originates primarily from diabetes-associated kidney disease [31]. In contrast, kidney transplantation did not restore urinary heparanase in our cohort, suggesting that heparanase is derived from distinct cell populations in diabetes and CKD conditions. In diabetes, urine heparanase correlated with blood glucose levels [31], suggesting that glucose is involved in heparanase regulation. Indeed, glucose has been shown to stimulate heparanase gene expression in rat glomerular epithelial cells and human embryonic kidney 293 cells, associating with decreased levels of surface HS [39]. In addition, glucose was found to stimulate secretion of heparanase by kidney and coronary artery endothelial cells [31], resulting in decreased intracellular content of heparanase [40]. Furthermore, high levels of glucose stimulated redistribution of heparanase-positive lysosomal vesicles in endothelial cells [40], resembling the effect of ATP [41] and insulin [31] on kidney 293 cells. Thus, elevated levels of heparanase found in diabetic patients may originate from enhanced secretion and/or expression of heparanase by kidney and endothelial cells. In transplanted patients, heparanase may originate from activated cells of the immune system residing in the circulation and kidney. Activated macrophages, neutrophils, CD4^+^ T cells, and mast cells exhibit heparanase activity which facilitates passage of immune cells into tissues and potentiates pro-inflammatory processes [42]. The presence of an inflammatory state in CKD patients facilitates renal function deterioration and is associated with adverse clinical outcomes [43]. It should be noted that the patients enrolled in this study had been transplanted one to ten years prior to heparanase determination and were likely to have developed an inflammatory condition to various extents. Pro-inflammatory state and consequently increased heparanase levels may be associated with the development of proteinuria and decreased renal function following transplantation. Studies examining the association between kidney inflammation and heparanase levels are currently in progress.

Experimental evidence supports the idea that heparanase-inhibiting heparin-derived agents and glycosaminoglycans favorably affect primary and secondary renal diseases, likely via inhibition of heparanase enzymatic activity. While a number of clinical studies have addressed the effect of these agents (i.e., sulodexide) in microalbuminuric and macroalbuminuric diabetic patients, very few have investigated their activity in non diabetic renal conditions [44], [45]. The present study implicates heparanase in the etiology of chronic renal allograft nephropathy, both as a circulating marker and as a drug target. Despite the introduction of novel drugs and the associated reduction in acute rejection rates, there has been no improvement in long-term allograft survival over the last decade. The newly described association between heparanase, proteinuria and decreased renal function is hoped to pave the way for new therapeutic options aimed at attenuating chronic renal allograft nephropathy, leading to improved graft survival and patient outcome.
